# Protective Effect and Mechanism of Total Flavones from *Rhododendron simsii* Planch on Endothelium-Dependent Dilatation and Hyperpolarization in Cerebral Ischemia-Reperfusion and Correlation to Hydrogen Sulphide Release in Rats

**DOI:** 10.1155/2014/904019

**Published:** 2014-06-22

**Authors:** Jun Han, Guo-Wei He, Zhi-Wu Chen

**Affiliations:** ^1^Department of Pharmacology, Wannan Medical College, Wuhu, Anhui 241002, China; ^2^The Affiliated Hospital of Hangzhou Normal University and Zhejiang University, Hangzhou, Zhejiang 310015, China; ^3^TEDA International Cardiovascular Hospital, Tianjin 300457, China; ^4^Department of Surgery, Oregon Health and Science University, Portland, OR 97239-3098, USA; ^5^Department of Pharmacology, Anhui Medical University, Hefei, Anhui 230032, China

## Abstract

We for the first time investigated the effect and mechanism of the total flavones of *Rhododendron simsii* Planch (TFR), a widely-used Chinese herb for a thousand years, on vasodilatation and hyperpolarization in middle cerebral artery (MCA) of rats subject to global cerebral ischemia-reperfusion (CIR). TFR (11~2700 mg/L) evoked dose-dependent vasodilation and hyperpolarization in MCA of both sham and CIR that were partially inhibited by 30 *μ*M N-nitro-L-arginine-methyl-ester and 10 *μ*M indomethacin and further attenuated by endogenous H_2_S synthese-CSE inhibitor PPG (100 *μ*M) or Ca^2+^-activated potassium channel (K_ca_) inhibitor TEA (1 mM). In whole-cell patch clamp recording, TFR remarkably enhanced the outward current that was inhibited by TEA. CIR increased CSE mRNA expression and the contents of H_2_S that were further increased by TFR. We conclude that, in MCA of CIR rats, TFR induces non-NO and non-PGI_2_-mediated effects of vasodilatation and hyperpolarization involving K_ca_ and increases CSE mRNA expression level in endothelial cells and H_2_S content in the cerebrum. These findings suggest that the response induced by TFR is potentially related to endothelium-derived hyperpolarizing factor mediated by the endogenous H_2_S and promote the use of TFR in protection of brain from ischemia-reperfusion injury.

## 1. Introduction

Ischemic cerebrovascular disease is one of the common entities threatening human health with high morbidity, disability rate, and mortality. Hunting for more effective drugs against cerebral ischemia-reperfusion injury (CIR) is one of the particular concerns in the pharmaceutical research.

Vascular tone plays an important role in the occurrence, development, and outcome of ischemic cerebrovascular disease. Endothelium-derived hyperpolarizing factor (EDHF) is the third kind of relaxing factor and autacoids [1, 2], described as the nonnitric oxide (NO) and nonprostaglandin I_2_ (PGI_2_) factor [3–7]. It induces the hyperpolarizing response and consecutive dilatation of the vascular smooth muscle cells by activating vascular calcium-activated potassium channels (K_ca_) [8]. Studies have indicated that EDHF is a modulator in regulating blood flow and vascular resistance during normal physiological states [9–12] and plays an even greater role following pathological conditions like organ ischemia, acidosis, and hypoxia [13, 14]. The role of EDHF has been demonstrated in several blood vessels, including mesenteric arteries [15, 16], coronary arteries [17, 18], carotid artery [19], femoral artery [20], and human arteries [12, 21]. However, the chemical nature of EDHF in cerebral arteries is still unclear. Others [22] and we [23] have found that the endogenous gas hydrogen sulphide (H_2_S) is related to the action of EDHF in cerebral arteries.

H_2_S is a gas signaling molecule and released from vascular endothelium. It has been shown that H_2_S could protect the heart from CIR damage in rats, leading to vasodilatation in rat aorta and mesenteric arteries [24, 25]. Furthermore, the effect of H_2_S-induced endothelium-derived dilatations was partly inhibited by antagonist of potassium ion channels [26, 27]. This indicates that H_2_S is potentially an EDHF candidate. Recent studies in our laboratory have found that H_2_S is possibly the EDHF in middle cerebral arteries (MCAs) from healthy and CIR rats [23, 28, 29].

Medicinal plants in nature may represent a desirable source to develop valid and safe drugs for treatment of diseases including ischemic brain injury. Flavonoids are effective ingredients in many Chinese herbs and exist extensively in natural plants. Flavonoids have various biological activities and pharmacological functions including vasorelaxing and anti-inflammatory effect as well as protective effects against CIR and free radical.

*Rhododendron simsii* Planch is a traditional Chinese medicine that has been used for a thousand years in China. The leaves of* Rhododendron simsii* Planch are used for the antitussive and expectorant effects as well as for the antiallergic and anti-inflammatory effects used as topical medicine. The root of* Rhododendron simsii* Planch, however, has been used for treatment of abnormal menstruation, diarrhea, cough, and exec [30]. These effects often lead the clinical application in treatment of chronic bronchitis, asthma, and rheumatic arthritis [30–32].

Total flavones of* Rhododendron simsii* Planch (TFR) are the active extract from the flowers of* Rhododendron simsii* Planch consisting of essential components of hyperion, quercetin, matteucinol, and rutin. The pharmacological effect of these components has been reported before. It has been shown that hyperin generates protective effects to attenuate the effect of free radicals, to reduce cerebral edema as well as cerebral vascular resistance, to increase cerebral blood flow, and to relax cerebral basilar artery in rats against cerebral ischemic injury [28, 33, 34]. In contrast, quercetin is reported to be able to inhibit pig platelet aggregation induced by thrombin through restraining Ca^2+^ influx in platelets [21] and protect from neuronal damage after transient global cerebral ischemia [35]. In addition, Qing et al. demonstrated that matteucinol could produce concentration-dependent vasodilatation in the isolated rat aorta, which was dependent on endothelium [36]. As to the role of rutin, it was reported to exert endothelium-dependent vasodilation by nitric oxide/guanylate cyclase (NO/GC) pathway and refrains platelet activating factor [37, 38]. Finally, as total flavones of* Rhododendron simsii* Planch, TFR has been found to have antispasmodic, analgesic, and anti-inflammatory roles [39].

Previous studies by others and us have shown that TFR has protective effects against cerebral ischemia-reperfusion injury [40] and that TFR may induce vascular dilatation in CBA and MCA from healthy rats [28, 29]. The present study was undertaken, for the first time, to investigate the underlying mechanisms of TFR against CIR in rats subject to global cerebral ischemia-reperfusion injury.

## 2. Methods

### 2.1. Drugs and Reagents

Total flavones of* Rhododendron simsii* Planch (TFR), content of flavones greater than 85%, were furnished by Hefei Heyuan Medicine Technology Limited Company (Hefei, China). N-Nitro-L-arginine-methyl-ester (L-NAME), indomethacin (Indo), DL-propargylglycine (PPG), tetraethylammonium (TEA), and collagenase were obtained from Sigma (St. Louis, MO, USA). Evans blue (EB) were purchased from Tiangen Biotechnology (Beijing, China). Mouse anti-rat CD31 FITC, anti-FITC microBeads, and Mini/Micli MACS Starting kit were purchased from Miltenyi Biotechnology (Auburn, CA, USA). PCR-Marker, RT-PCR test kit, RNAiso Plus, CSE, and *β*-actin primer were purchased from Baoshengwu Biological Company (Dalian, China). Diethyl pyrocarbonate (DEPC) was purchased from Solarbio. Fetal bovine serum was purchased from Hyclone (Logan, UT, USA). Phosphate saline solution (PSS) [41] including (in mM) 118 NaCl, 3.4 KCl, 2.5 CaCl_2_, 1.2 KH_2_PO_4_, 1.2 MgSO_4_, 25 NaHCO_3_, and 11.1 glucose was effervesced with 95% O_2_ and 5% CO_2_, pH adjusted to 7.4 with NaOH. The PSS solution was always oxygenated during the latency period.

### 2.2. Animals and Experimental Protocol

Male Sprague-Dawley rats weighing 250–350 g were provided by the Experimental Animal Center of Anhui Medical University (Hefei, China) (Certificate number SCXK 2005-001). All animal studies and surgical procedures were conformed to the regulations defined by the Animal Care Committee of Anhui Medical University, which is in line with the Guide for the Care and Use of laboratory Animals published by the US National Institute of Health (NIH publication number 85-23, revised 1996).

All animals were fed with free access to food and tap water in clean plexiglass cages with stainless steel wire lids and filter tops at a controlled temperature 22 ± 3°C in room and kept with a 12 hours light/dark cycles. Rats were used in the experiment during the photostage of the light/dark cycles.

For RT-PCR test, rats were divided into the 6 groups (*n* = 8 for each), that is, sham, NS (control with treatment of normal saline), TFR 25 mg/kg, TFR 50 mg/kg, TFR 100 mg/kg, TFR 100 mg/kg, and PPG 37.5 mg/kg, and the animals were administered via tail vein injection at 20 min before ischemia.

In ELIASA experiment, rats were allocated to 5 groups (*n* = 8 for each), that is, sham, NS, TFR 25 mg/kg, TFR 50 mg/kg, and TFR 100 mg/kg and were treated via tail vein injection at 20 min before ischemia.

### 2.3. Extraction of Total Flavones from* Rhododendron simsii* Planch

A large number of components may be identified from* Rhododendron simsii* Planch [42]. In brief, the dried flowers of* Rhododendron simsii* Planch (500 g × 3) were dunked into 75% alcohol for 8 hours and then boiled under reflux for 2 hours. After being filtered, the boiled liquid was concentrated. The concentration was chromatographed on polyamide thread columns (25 × 18 cm; Wako Pure Chemical Industry Co., Osaka, Japan) to procure crude total flavones, which were further depurated by ethyl acetate and ethanol (V : V 76 : 38). The final freeze-dried yellow powder of total flavones was acquired and utilized for this study. UV-spectrophotometry was used to determine the content of TFR.

### 2.4. Global Cerebral Ischemia-Reperfusion Rat Models

Rat models of CIR were established by the modified method of Pulsinelli-Brierley (four-vessel occlusion, 4-VO) [43, 44]. In brief, rats were anesthetized with 10% chloral hydrate (0.3–0.35 g/kg body wt, ip) (Shanghai, China). The bilateral vertebral arteries were electrocoagulated and the incision was managed with suturing, followed by exposing and isolating the bilateral common carotids. All animals were fed in rearing cages for 24 h with adlibitum feeding. In the experiment, bilateral carotid arteries were simultaneously clamped by using two nontraumatic arteriole clips with keeping the core body temperature at 37°C. After 0.5 h of ischemia, the microvascular clamps were gently removed to recover cerebral blood flow through bilateral carotid arteries. The eligible experimental animals presented stiffness of the forepaws and loss of righting reflex during ischemia and survived after 0.5 h of ischemia and 2 h of reperfusion. In the sham-operation group, vertebral arteries were not electrocoagulated and carotid arties were not occluded.

Electroencephalogram (EEG; EB Neuro Corp., S.p.A, Firenze, Italy) was recorded at 5 min before and 0.5 h after ischemia and reperfusion by 5 min, 15 min, 30 min, 45 min, 60 min, and 120 min, respectively.

### 2.5. Vascular Experiments

Rats were decapitated after anesthetizing with 10% chloral hydrate via peritoneal injection. The brain was fleetly removed from the pericranium and immersed in ice-cold physiological salt solution (PSS). Then the MCA was carefully dissected, made into segments of 0.6–0.8 cm in length, and placed in a vessel container. The end of each MCA segment was inserted into two glass micropipettes and kept within a vessel chamber pressurized to a mean of 85 mmHg to establish a flow discharge of 150 *μ*L/min through the lumen. The vessel was bathed with PSS aerated with 95% O_2_-5% CO_2_ (PH 7.3–7.5) and kept at 37°C using a fixed heat-exchanger device. After MCA segment was mounted, the container was placed on the stage of a stereoscopic microscope (Bengbu, China) equipped with a digital camera (Nikon, Tokyo) and a computer screen. The reproducible constriction was acquired by adding 30 mM KCl to the superfusate. L-NAME (30 *μ*M) and Indo (10 *μ*M) were utilized to inhibit the products of NO synthase and cyclooxygenase in experiments, respectively. Outer diameter of each vessel was measured directly from the video screen (magnification of ×100). The average values of maximum and minimum diameters were recorded on MCA during vasomotion. MCA dilatations were represented as the percentage of the maximum diameter (% *D*_max⁡_) using the following equation:

(1) $$\begin{array}{c} \%\;\;\text{Dilatation}=\left[\frac{\left(D-D_{\min}\right)}{\left(D_{\max}-D_{\min}\right)}\right]\cdot 100, \end{array}$$

where *D* represents the vessel diameter after luminal administration of the reagents, either TFR or L-NAME, and so forth, *D*_min⁡_ expresses the diameter after the addition of 30 mM KCl, and *D*_max⁡_ stands for the maximum diameter obtained at 85 mmHg luminal pressure after 1 h equilibrium.

Mechanical method is used to remove vascular endothelial cell of MCA [45]. Briefly, a slimsy hairline of head is utilized to rub back and forth into lumens of MCA for removal vascular endothelial cell. Ach (10^−5^ mol/L) was used to validate the integrity of endothelium and usually it was less than 10% [46]. As suggested, it is considered that endothelium is completely removed when ACh-induced relaxation is less than 30% [47].

### 2.6. Microelectrode Experiments

**Figure 1:**
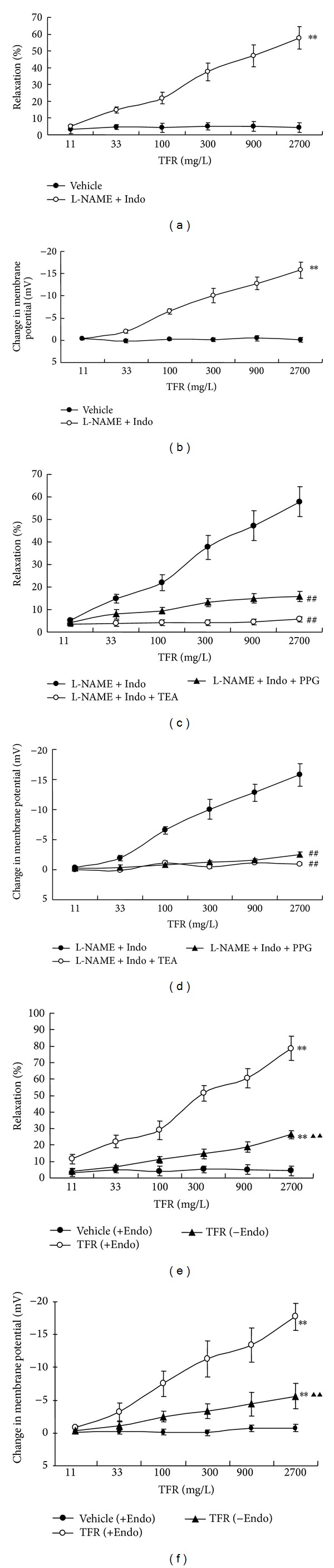
The dilatation (a) and hyperpolarization (b) of middle cerebral arteries of rats subjected to cerebral ischemia-reperfusion to TFR in the presence of 30 *μ*M L-NAME (an inhibitor of nitric oxide synthase) and 10 *μ*M Indo (an inhibitor of cyclooxygenase); effect of TEA (1 mM, an inhibitor of Ca^2+^-activated potassium channel) or PPG (100 *μ*M, an inhibitor of cystathionine-*γ*-lyase) on non-NO and non-PGI_2_-mediated vasodilation (c) and hyperpolarization (d) elicited by TFR in VSMC from middle cerebral artery of rats subjected to cerebral ischemia-reperfusion; effect of endothelial denudation on the dilatation (e) and hyperpolarization (f) to TFR in middle cerebral arteries of rats subjected to cerebral ischemia reperfusion. Values are presented as means ± SE. +Endo: endothelium-intact and −Endo: endothelium-denudated. **P < 0.01 compared with vehicle group; ^##^ *P* < 0.01 compared with L-NAME+Indo group; ^▲▲^ *P* < 0.01 compared with TFR (+Endo) group. Comparisons were performed by unpaired Student's *t*-test.

Isolated MCA segments for sham operation and CIR vessels were longitudinally incised, fixed within 10 mL silica gel slot (a mean pressure of 85 mmHg), superfused with PSS, maintained at 37°C, and oxygenated with 95% O_2_-5% CO_2_. In some experiments, superfusate was filled with 30 *μ*M L-NAME plus 10 *μ*M Indo. Vascular smooth muscle cells (VSMCs) of the MCA were impaled by microelectrodes surveyed intracellular membrane potential (*E*_*m*_) as previously reported. In this experiment, KCl was deleted from those special protocols as it could lead to vasoconstriction with the consequent tissue motion being disadvantageous to keeping intracellular membrane potential recordings. Each VSMC was pierced by using glass microelectrodes perfused with 3 M KCl (electrode resistances ranged from 40 to 80 MΩ). The typical pierce was successfully emerged a sudden drop in *E*_*m*_ kept stable for at least 2 min before initiating the experiment. A single *E*_*m*_ value for each condition in a specified MCA was acquired by averaging four to six different VSMC impalements. The conventional high impedance amplifier (Intra 767; World Precision Instruments, Sarasota, FL, USA) was utilized to record the potential difference and interference (50 Hz) at the amplifier output that was selectively moved aside. The MacLab system connected with Chart 5 software (AD Instruments, Castle Hill, NSW, Australia) was used to monitor and analyze *E*_*m*_. The *n* value of Figure 1 refers to the number of experimental rats, which is not the number of impalements.

### 2.7. Whole Cell Patch Clamp Recording Experiments

The acute separation of MCA smooth muscle cells in rats has been described elsewhere. Briefly, Rats were killed by detruncation and MCA was cleanly isolated and placed in PSS containing 1.0 mg/mL Type II collagenase and 0.5 mg/mL papain to incubate at 37°C for 35 min. Mechanical separation of the digested tissue into a single vascular smooth muscle cell and the formation of cell suspension were performed by using polished Pasteur pipettes. The cell was plated onto glass coverslips and incubated for 45 min prior to electrophysiological study and left to stick to the glass coverslip before an experiment was started.

Electrophysiological measurements from MCA smooth muscle cells were made using the whole cell patch clamp recording technique. Whole cell currents were made using an EPC-10 amplifier and pulse software. The membrane currents were filtered at 1 kHz and stored. When starting each experiment, the junction potential between the bath solution and pipette solution was correctly adjusted to zero. Test pulses were performed with a 10 mV increase from −60 to 100 mV with a holding potential of −60 mV for 500 ms. The patch pipettes were drawn out of borosilicate glass (resistance 3 to 5 MΩ, P-97-type microelectrode puller instrument from Sutter, USA). The bath solution for recording K_ca_ current was composed of (in mM) 140 NaCl, 1 MgCl_2_, 5 H-HEPES, 1 CaCl_2_, 5 KCl, and 10 glucose (pH adjusted to 7.4 with NaOH). The recording pipette solution contained (in mM) 105 K-gluconate, 1 MgCl_2_, l30 KCl, 10 H-HEPES, 2.1 CaCl_2_, and 5 Na_2_ATP; pH was set to 7.2 with NaOH. Cells were continuously superfused with the bath solution containing testing chemicals. The recording values of the current were shown by the current density (pA/pF) and the experimental results analyzed and mapped using Igor 5 software.

### 2.8. Reverse Transcription-Polymerase Chain Reaction (RT-PCR)

The endothelial cells from MCA of rats were isolated and purified using magnetic activated cell sorting (MACS) by the method described by DeLeve et al. and Kader et al. All reagents for MACS separation were purchased from Miltenyi Biotec (Bergisch Gladbach, Germany). In brief, 10 *μ*L FITC was incubated in 100 *μ*L buffer (35~40 min at 4°C) and added in 2 mL phosphate saline solution (PSS) and centrifugated for 10 min (1000 rpm). The endothelial cell of middle cerebral artery cells in rats was marked by using mouse anti-rat CD31 FITC. Anti-FITC microBeads (10 *μ*L) were incubated with 10^7^ cells per mL for 30 min (4~8°C). Endotheliocytes of MCA (positive cells) in rats were separated and purified by utilizing a magnetic separation column, which was washed 3× with PSS. A total of 10^5^ cells/mL were placed in cryogenic vial and maintained in nitrogen canister for reserve. The level of endotheliocyte CSE mRNA was determined by semiquantitative reverse transcription (RT)-PCR [48]. Total RNA was isolated using RNA isolation kit (Baoshengwu, Dalian, China). Extraction of RNA was successful, and the RNA integrity and quantity were estimated ordinarily by absorbability (A260/A280: 1.8~2.0). Ethidium bromide fluorescence of RNA was separated by electrophoretic technique on 1% tetrabromoethane (TBE) containing agarose gels. A standardized semiquantitative RT-PCR method was utilized on amplification of the target genes and *β*-actin, a constitutively expressed gene, as reference gene. The sequence specific primers for the RT-PCR were used as follows (Baoshengwu, Dalian, China): (forward) 5′-CCACCACAACGATTACCCA-3′, (reverse) 5′-TCAGCACCCAGAGCCAAAG-3′, and 334 bp for MCA CSE and (forward) 5′-CTGTCCCTGTATGCCTCTG-3′, (reverse) 5′-ATGTCACGCACGATTTCC-3′, and 236 bp for *β*-actin. Sample RNA 1 *μ*L was reversely transcribed in 10 *μ*L of a solution including 1 *μ*L of dNTP mixture (10 mM each), 1 *μ*L of oligo dT primer (2.5 *μ*M), and RNase free dH_2_O. The mixture was heated to 65°C for 5 min for RNA denaturation and cooled quickly to 4°C. Next, 4 *μ*L of 5× primescript buffer, 0.5 *μ*L of RNase inhibitor (40 U/*μ*L), 0.5 *μ*L of primescript RTase (for 2 step), and 5 *μ*L of RNase free dH_2_O mix were added, and the reaction was performed in following conditions: 30°C for 10 min, 42°C for 30 min, 95°C for 5 min, and 4°C for 5 min. Then the RNA obtained was amplified by PCR in total of 35 cycles (2 min at 94°C, 30 s at 50°C/53°C, and 45 s at 72°C). *β*-Actin was utilized as an internal control. After amplification, each PCR product (5 *μ*L) was analyzed by electrophoresis on a 1% agarose gel in 1×TBE buffer, stained with ethidium bromide, and photographed under ultraviolet light with a multi-image light cabinet.

### 2.9. Measurement of Endogenous H_2_S Production

Tissue H_2_S production rate was determined as described previously [49] with modifications. In brief, the right cerebral cortex of rats killed by detruncation was stored in liquid nitrogen and homogenized 12% (w/v) tissue homogenate in 50 mM ice-cold potassium phosphate buffer solution (pH 8.0), and then the homogenate was centrifugated for 10 min (47000 ×g, 4°C). The pipette was used to move homogenate (75 *μ*L) into another a centrifugal tube in which 1% zinc acetate (0.25 mL) and distilled water (0.45 mL) were added, respectively. After incubating for 10 min, 0.25 mL of 10% three chloroacetic acid was added into the reaction mixture to stop the reaction, followed by second 10-min centrifugation (14000 ×g, 4°C). The supernate in the centrifugal tub was then transferred to test tubes. Subsequently, 133 *μ*L of 20 mM N,N-dimethyl-p-phenylenediamine sulphate in 7.2 M HCl was added immediately, followed by adding 133 *μ*L of 30 mM FeCl_3_ in 1.2 M HCl. The absorbance of the resulting solution at 670 nm was measured 20 min later with an automatic ELISA (BioTEK, USA). The content of H_2_S was calculated by the calibration curve of the standard H_2_S solutions.

### 2.10. Statistical Analysis

All data are expressed as the mean ± standard deviation of the mean. Quantitative analysis was estimated by using one-way analysis of variance (ANOVA) with Bonferroni's posttest. The differences between sham and CIR vessels were tested using the unpaired Student's *t*-test. A probability (*P*) value <0.05 was considered as statistically significant. All analyses were performed using the Statistical Package for Social Sciences (SPSS) 11.5 software.

## 3. Results

### 3.1. Effect of L-NAME+Indo on TFR-Induced Vasodilatation in CIR Vessels

Figure 1 shows that in vessels precontracted by 30 mM KCl, after treatment with 30 *μ*M L-NAME and 10 *μ*M Indo, TFR (11~2700 mg/L) evoked dose-dependent vasodilation (the percentage of maximal dilatation, *E*_max⁡_: 57.8 ± 6.6%, Figure 1(a)) and hyperpolarization (the maximal change in *E*_*m*_: −15.8 ±1.9 mV, Figure 1(b)) in MCA of CIR rats (*P* < 0.01, versus Vehicle).

### 3.2. Combined Effect of L-NAME+Indo+TEA on TFR-Induced Dilation in CIR Vessels

TEA (1 mM), an inhibitor of Ca^2+^-activated potassium channel, markedly attenuated TFR-elicited non-NO and non-PGI_2_ dilatation (57.8 ± 6.6% versus 5.7 ± 1.2%, *P* < 0.01, Figure 1(c)) and hyperpolarization (−15.8 ± 1.9 mV versus −0.9 ± 0.2 mV, *P* < 0.01, Figure 1(d)) in the presence of L-NAME (30 *μ*M) plus Indo (10 *μ*M). These data revealed that the TFR-mediated non-NO, non-PGI_2_ effects were potentially associated with calcium-activated potassium channels.

### 3.3. Effect of L-NAME+Indo+PPG on TFR-Induced Dilatation in CIR Vessels

After inhibition of NO and PGI_2_ synthase with 30 *μ*M L-NAME and 10 *μ*M Indo, TFR-induced vasodilatation and hyperpolarization were notably inhibited by 100 *μ*M PPG, a blocker of endogenous H_2_S synthese-cystathionine-*γ*-lyase (CSE), resulting in reduced dilatation (57.8 ± 6.6% versus 15.8 ± 2.4%, Figure 1(c)) and hyperpolarization (−15.8 ± 1.9 mV versus −2.5 ± 0.4 mV, *P* < 0.01, Figure 1(d)). These findings indicated that TFR-induced non-NO and non-PGI_2_ mediated effects were potentially related to the endogenic release of H_2_S.

### 3.4. Effect of Endothelial Denudation on TFR-Induced Vasodilatation in CIR Vessels

The TFR (11~2700 mg/L) induced vasodilatation and hyperpolarization were significantly attenuated by removal of endothelium in MCA of CIR rat. In fact, in vessels precontracted by 30 mM KCl, the maximal dilatation reduced from 78.6 ± 7.5% to 26.4 ± 2.5% (Figure 1(e)). Similarly, the maximal hyperpolarization reduced from −17.7 ± 2.1 mV to −5.6 ± 1.9 mV (Figure 1(f)).

### 3.5. Effect of TFR on Calcium-Dependent Potassium Current in VSMC from MCA of CIR Rat

**Figure 2:**
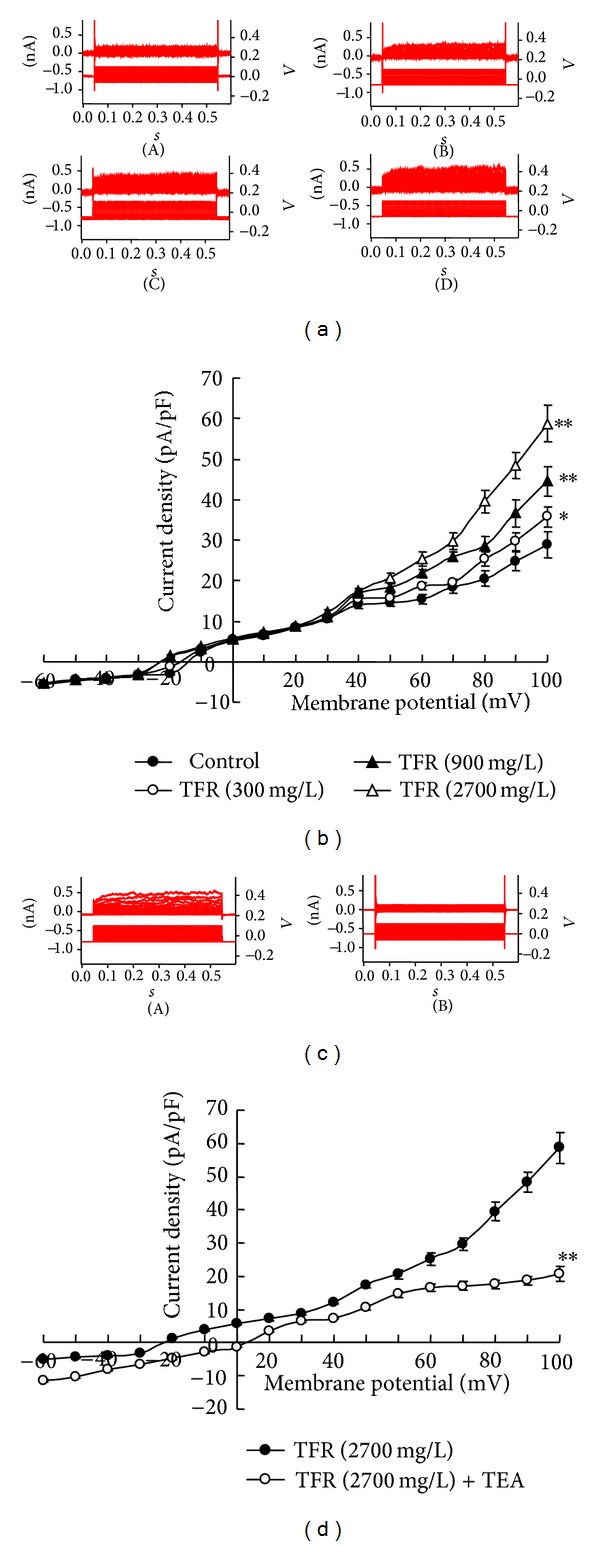
The enhancement of TFR on the outward current in VSMC from middle cerebral arteries of rats subjected to global cerebral ischemia-reperfusion. (a) The trace of outward current in one VSMC, A: control, B ~ D: 300,900 and 2700 mg/L TFR and (b) the curve of current-voltage relationship. (*n* = 6); effect of TEA on the TFR-induced enhancement of outward current in VSMC from middle cerebral artery of rats subjected to global cerebral ischemia-reperfusion. (c) The trace of outward potassium current in one VSMC. A: TFR 2700 mg/L, B: TEA 1 mM + TFR 2700 mg/L and (d) the curve of current-voltage relationship. *n* = 6. Values are presented as means ± SE. *P < 0.05, **P < 0.01, compared with control group. **P < 0.01 compared with TFR (2700 mg/L) group. Comparisons were performed by unpaired Student's *t*-test.

The outward current shown in Figure 2 was induced in VSMC from MCA of rats subject to CIR. At 300–2700 mg/L, TFR remarkably enhanced the outward current and the enhancement was inhibited by 1 mM TEA, an inhibitor of K_ca_ channel. The results suggested that the enhanced effect of TFR on calcium-dependent potassium current in VSMC from MCA of CIR rats involves the opening of K_ca_ channels.

### 3.6. Effect of TFR on the Level of CSE mRNA Expression in MCA Endothelial Cells of CIR Rats

**Figure 3:**
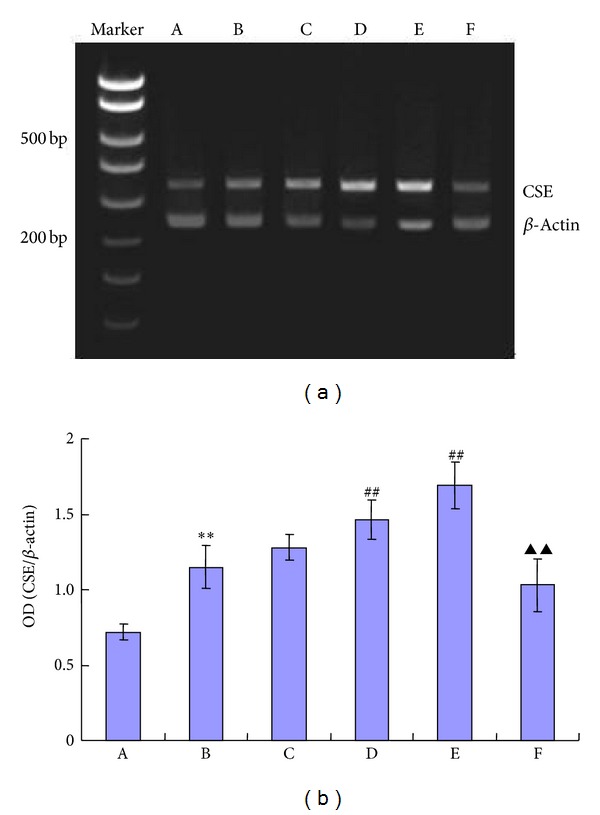
Effects of TFR on the levels of endothelium cells of middle cerebral artery CSE mRNA expression in rats subjected to cerebral ischemia-reperfusion. A: sham, B: NS, C~E: 25, 50, and 100 mg/kg TFR, and F: 37.5 mg/kg PPG + 100 mg/kg TFR. All values are presented as means ± SE. All comparisons were assessed by unpaired Student's *t*-test. **P < 0.01 compared with sham group, ^##^ *P* < 0.01 compared with NS group, and ^▲▲^ *P* < 0.01 compared with TFR (100 mg/kg) group.

The level of CSE mRNA expression in the purified and isolated endothelial cells from MCA of CIR rats was increased (*P* < 0.01, versus control, Figure 3). TFR (50, 100 mg/kg) markedly potentiated the CSE mRNA expression, which was upregulated in the endothelial cells from MCA of CIR rats (*P* < 0.01, versus NS, Figure 3).

### 3.7. Effect of TFR on H_2_S Content in the Cerebrum of CIR Rats

The contents of H_2_S were significantly increased in the cerebrum of rats subject to CIR and pretreatment with TFR in the range of 25 to 100 mg/kg further increased the H_2_S contents (*P* < 0.05).

## 4. Discussion

In this study, we have found that (1) TFR induces endothelium-dependent vasodilatation and hyperpolarization in MCA of CIR rat; (2) the non-NO and non-PGI_2_-mediated effects of vasodilatation and hyperpolarization of VSMCs induced by TFR involves K_ca_ channels and therefore the response is related to EDHF; and (3) TFR increases the level of CSE mRNA expression in MCA endothelial cells and H_2_S content in the cerebrum of CIR rats.

### 4.1. TFR Induced Endothelium-Dependent and Endothelium-Independent Responses

The present study showed that 11~2700 mg/L TFR-induced vasodilatation and hyperpolarization have endothelium-dependent and endothelium-independent components in MCA of CIR rats. The endothelium-dependent component is reflected by the significant attenuation of the response by removal of the endothelium (Figures 1(e) and 1(f)). The endothelium-independent component is reflected by the remaining response after removal of the endothelium (Figures 1(e) and 1(f)).

### 4.2. TFR Induced EDHF-Mediated Dilatation and Hyperpolarization of the VSMCs

In the present study, we found that in MCA of CIR rats with blockage of NO and PGI_2_ formation with combination of L-NAME and Indo, TFR (11~2700 mg/L) induced concentration-dependent vasodilatation and hyperpolarization of smooth muscle cells, indicating that TFR may induce non-NO, non-PGI_2_-mediated vasodilatation and hyperpolarization. It is likely that TFR-mediated cerebrovascular dilatation may be related to the endothelium-dependent vasodilatation of hyperin, matteucinol, and rutin in TFR that were demonstrated previously [28, 36, 37]. The present study further demonstrated that these responses involve the activation of K_ca_ channels. As is well known now, EDHF opens K_ca_ channels on the smooth muscle cell and induces hyperpolarization of smooth muscle cell membrane potential, resulting in reduced Ca^2+^ influx and vascular dilatation [9, 50–52]. Therefore, an important characteristic of EDHF response is that it may be inhibited by K_ca_ channel blockers.

In acutely isolated smooth muscle cell of MCA in rats, we found that the calcium-dependent potassium current of the whole cell in both control and TFR groups exhibited a voltage-dependent manner, significantly and outwardly rectifying characteristics in high voltage and that the outward current density induced by TFR (300~2700 mg/L) was significantly enhanced. This was abolished by 1 mM TEA, indicating that the outward current was calcium-activated potassium current and TFR directly enhanced this current in smooth muscle cells from MCA in CIR rats.

Previous studies have shown that 1 mM TEA, nonselective potassium channel blocker, specifically blocks the opening of K_ca_ channels [9, 27, 53]. Indeed, we found that 1 mM TEA almost abolished the non-NO non-PGI_2_-mediated vasodilation and hyperpolarization induced by TFR in the presence of L-NAME and Indo in MCA of CIR rats. These results indicate that the effects of TFR involve the opening of K_ca_ channels and this finding clearly shows that TFR-induced non-NO non-PGI_2_ effects are related to EDHF.

### 4.3. EDHF-Mediated Responses in Rat Cerebral Vessels Is Related to H_2_S

Studies from others and ourselves have suggested that H_2_S induces hyperpolarization of smooth muscle cells and vasodilatation [23, 24, 53] and H_2_S modulates the functions of the central nervous system by regulating vascular smooth muscle tone and cerebral blood supply [25]. In the cardiovascular system, CSE is considered to be the only enzyme to produce H_2_S and is expressed in vascular smooth muscle cells and endothelial cells, with endothelial cells being more prominent [54]. We have proposed that H_2_S produced in vascular endothelium of CBA of rats may be EDHF [23].

In the present study, 100 *μ*M PPG, an inhibitor of endogenous H_2_S synthese-cystathionine-*γ*-lyase (CSE), combined with L-NAME and Indo, eliminated EDHF-mediated vasodilatation and hyperpolarization of smooth muscle cell induced by TFR in MCA of CIR rats, suggesting that TFR- induced EDHF effects are associated with the production of endogenous H_2_S. Further, our RT-PCR experiments by using endothelial cells from MCA in CIR rats separated and purified by the magnetic activated cell sorting technique (MACS), currently the most advanced technology on cell separation [55], demonstrated the expression of CSE mRNA, indicating that the synthesis of endogenous H_2_S enzymes is expressed in endothelial cells from MCA. In addition, the endothelial cells CSE mRNA expression was significantly higher than that of sham group, indicating that CIR promotes endothelial cells of MCA to synthesize CSE and increases the generation of H_2_S. This is in accordance with our previous experimental results [23]. We speculate that the increase of CSE mRNA expression and H_2_S production of the endothelial cells from MCA might serve as a compensative protection to reserve endothelium-derived vasodilatation in CIR rats. Such protective and vasodilator effect is beneficial to the vessel subject to ischemia-reperfusion injury.

The present study also demonstrated that the content of H_2_S in the brain tissue was significantly higher after ischemia-reperfusion (compare NS and sham, *P* < 0.01) and this increase was further spurred by pretreatment of TFR (25–100 mg/kg) (Table 1).

Interestingly, the present study demonstrated for the first time that TFR may increase the CSE expression levels of endothelial cells of MCA in CIR rats and therefore may increase the synthesis of endogenous H_2_S that results in vasodilatation effect in cerebrovascular system against brain ischemia-reperfusion injury. This result is in accordance with previous reports that showed the protective effects against cerebral ischemia by hyperin and quercetin, which are primarily active constituents in TFR [28, 35]. The findings from our study, together with previous reports, may promote the development and utilization of TFR as the new strategy in management of ischemia-reperfusion injury.

In summary, we for the first time found that in MCA of CIR rats, TFR induces the non-NO and non-PGI_2_-mediated effects of vasodilatation and hyperpolarization of VSMCs, involving K_ca_ channels, and increases the level of CSE mRNA expression in MCA endothelial cells and H_2_S content in the cerebrum of CIR rat. This finding suggests that the non-NO and non-PGI_2_ response induced by TFR is potentially related to EDHF, mediated by the endogenous H_2_S. Therefore, our study promotes the use of Chinese medicine in protection of brain and may form a new strategy in prevention and treatment of ischemia-reperfusion injury by using TFR.

## Figures and Tables

**Table 1:** Effect of TFR on cerebral H_2_S level in rats subjected to cerebral ischemia-reperfusion.

Group	Dose (mg/kg)	*n*	H_2_S (nmol/g)
Sham	—	8	11.5 ± 1.1
NS	—	8	17.6 ± 1.6∗∗
TFR	25	8	19.8 ± 1.7^∗∗#^
	50	8	22.0 ± 2.3^∗∗##^
	100	8	25.6 ± 2.5^∗∗##^

**P < 0.01 compared to the sham control, ^#^ *P* < 0.05, ^##^ *P* < 0.01 compared to the NS control. Each group consisted of 8 rats. TFR: total flavones of *Rhododendron simsii* Planch, Sham: sham operation control group, and NS: normal saline control group.

## Abbreviations

TFR: Total flavones of* Rhododendron simsii* Planch
CIR: Cerebral ischemia-reperfusion
EDHF: Endothelium-derived hyperpolarizing factor
H_2_S: Hydrogen sulfide
K_ca_: Calcium-activated potassium channel
CSE: Cystathionine-*γ*-lyase.
